# Attitudes of Patients Toward Adoption of 3D Technology in Pain Assessment: Qualitative Perspective

**DOI:** 10.2196/jmir.2427

**Published:** 2013-04-10

**Authors:** Fotios Spyridonis, Gheorghita Ghinea, Andrew O Frank

**Affiliations:** ^1^Brunel UniversityDepartment of Information Systems and ComputingUxbridgeUnited Kingdom; ^2^Brunel UniversityCentre for Research in RehabilitationSchool of Health Studies and Social CareUxbridgeUnited Kingdom

**Keywords:** pain assessment, 3-dimensional image, health care systems, health care delivery, patient acceptance of health care, qualitative research

## Abstract

**Background:**

Past research has revealed that insufficient pain assessment could, and often, has negative implications on the provision of quality health care. While current available clinical approaches have proven to be valid interventions, they are expensive and can often fail in providing efficient pain measurements. The increase in the prevalence of pain calls for more intuitive pain assessment solutions. Computerized alternatives have already been proposed both in the literature and in commerce, but may lack essential qualities such as accuracy of the collected clinical information and effective patient-clinician interaction. In response to this concern, 3-dimensional (3D) technology could become the innovative intervention needed to support and improve the pain assessment process.

**Objective:**

The purpose of this analysis was to describe qualitative findings from a study which was designed to explore patients’ perceptions of adopting 3D technology in the assessment of their pain experience related to important themes that might positively or negatively influence the quality of the pain assessment process.

**Methods:**

The perceptions of 60 individuals with some form of pain in the area of Greater London were collected through semi-structured interviews. Of the 60 respondents, 24 (43%) produced usable responses and were analyzed for content using principles of the grounded theory approach and thematic analysis, in order to gain insight into the participants’ beliefs and attitudes towards adopting 3D technology in pain assessment.

**Results:**

The analysis identified 4 high-level core themes that were representative of the participants’ responses. These themes indicated that most respondents valued “the potential of 3D technology to facilitate better assessment of pain” as the most useful outcome of adopting a 3D approach. Respondents also expressed their opinions on the usability of the 3D approach, with no important concerns reported about its perceived ease of use. Our findings finally, showed that respondents appreciated the perceived clinical utility of the proposed approach, which could further have an influence on their intention to use it.

**Conclusions:**

These findings highlighted factors that are seen as essential for improving the assessment of pain, and demonstrated the need for a strong focus on patient-clinician communication. The participants of this analysis believed that the introduction of 3D technology in the process might be a useful mechanism for such a positive health care outcome. The study’s findings could also be used to make recommendations concerning the potential for inclusion of 3D technology in current clinical pain tools for the purpose of improving the quality of health care.

## Introduction

### Overview

**Figure 1:**
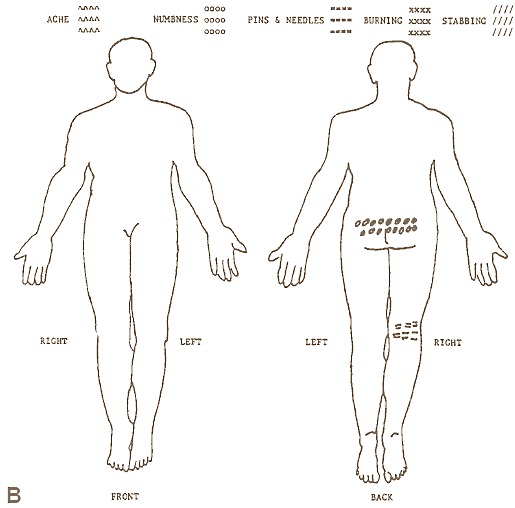
A completed pain drawing [24].

In medical practice, high quality clinical information is essential in providing high quality health care. When assessing a patient for pain, the only valued information that can typically be used are suggestive descriptions or self-reports from the patient, which are considered as the best available source of information for pain measurement [1]. These, however, are also considered to be subjective in nature [2], as they are highly influenced by a variety of psychological and cultural factors [3]. In fact, it is not uncommon in some patients that psychological problems may have actually caused some of the pain by adding stress to the body, or the stress of the pain may have caused psychological problems [4]. As a result, the ability of a clinician to provide a wholly accurate diagnosis can be, and often is, undermined due to inaccurate pain descriptions from the patient. In response to this, clinicians in primary care have been using the “pain drawing” shown in Figure 1 as a means to collect and manage pain information of higher quality.

### The Pain Drawing in the Measurement of Pain

The pain drawing has long been applied to the field of pain documentation [5-9], offering patients the ability to self-record information about the *location* and *type* of pain they are experiencing on a paper-based diagram of a human figure. Such a topographical representation of pain is argued to be very useful in summarizing patients’ description of their pain in an interpretable way for clinicians [5], and can be further used to better monitor change in patients’ pain situation [10]. The patients typically mark the location of pain experienced on a blank human diagram, using either specific symbols [11] or colours [12], in order to indicate various pain types.

As a result of its potential, this tool has been employed over the years to assess a variety of pain-related conditions ranging from simple back pain to more serious medical problems. In some instances, the pain drawing was the most used tool to evaluate the location of back pain [13], and in other instances, the pain drawing was most commonly used to evaluate pain as a result of knee osteoarthritis [14] and to examine the association of depression with spinal stenosis surgery [15].

Nevertheless, the results from a pan-European consensus report [16] showed that one in five Europeans (19%) was estimated to suffer from pain, a figure that seems to indicate that there is still an overall lack of success in its assessment, despite the benefits of the pain drawing for the intended purpose. This might be an indication of underdeveloped clinical tools and limited on-going research with respect to improving them. This same report particularly highlighted that the percentage of 2019 people in 15 European countries whose pain was still not adequately managed was 38%, and suggested that this was indeed partially owed to the inappropriate and ineffective management and treatment of pain, which needs to be improved.

Accordingly, in the pain literature, recent studies similarly revealed that this situation is existent, evidencing that one of the most important pain measurement tools, the pain drawing, has indeed remained highly underdeveloped. In particular, it is noteworthy that in such studies (eg, [9,17-22] the same paper-based, 2-dimensional (2D) human diagram is still being used as the main means to report and show the location of pain that can typically occur in any part of our 3-dimensional (3D) human body.

This is certainly one of the biggest drawbacks of the pain drawing, as it implies serious concerns about its performance when it comes to accurately visualizing patients’ pain descriptions. For instance, it is very common for pain to occur on the inside of a thigh, a location that is not easily captured in 2D pain drawings. It is therefore expected that a pain reporting mismatch is present in the current version of the pain drawing, resulting in (1) patients being unable to accurately report the pain that they are experiencing, and (2) making the assessment a time-consuming process with possible irrelevant medical data collected that can lead to the ineffective management of pain.

Results from a number of various studies in the area of information visualization seem to consent that 2D visualization is indeed not anymore useful for a complete understanding of the “object” under investigation, mainly because it lacks the natural depth cues (eg, perspective, shading, and occlusion) [23]. As such, notwithstanding its advantages, it is essential for new interventions for more accurate pain assessment to be developed.

### The Need for Adopting 3D Technology

With the emergence of 3D technology, the field of health care has already adopted 3D technology for a variety of uses, and it has become one of the most common methods for visualizing medical information. In the area of information technology, the 3D concept is used to describe a real or imagined environment that can be experienced visually in the 3 dimensions of width, height, and depth, and that may additionally provide an interactive experience. According to [24], 3D technology offers significant benefits over 2D, in particular: (1) displaying data in 3D can make it easier for users to manipulate the data, (2) improvement of the understanding of 3D structures when users have the ability to manipulate it, and (3) 3D makes it possible to make the layout of a designed object more consistent with its intended role and visualize it as perceived in its natural environment.

Along these lines, researchers have used 3D computer reconstructions to evaluate the pathology of a spinal cord injury [25] and to construct 3D virtual images from computerized medical scans [26]. In both examples, 3D technology was extremely beneficial because the models produced could be observed from many different viewpoints, while rotation and zooming features were combined to allow observer navigation within the tissue of interest. Such feature benefits were anticipated to provide and improve the depth cues that 2D pain drawings currently lack.

As opposed to a variety of other areas in health care, until recently, the traditional 2D pain drawings had never caught up with 3D technology, thus, it had always been lacking the benefits mentioned above. Previous work [2,27] attempted to address this issue by introducing a novel 3D pain drawing that employed the aforementioned features in the effort to provide better and more accurate measurements of pain. This new pain drawing was evaluated for its *usability* and *user satisfaction* in the self-reporting of pain by different groups of patients suffering from pain, producing very positive outcomes.

While clinicians seem to be embracing 3D technology to support a wide range of generic clinical activities, it is unclear in the literature how the category of patients suffering from pain would actually perceive the adoption of 3D technology in everyday practice for the intended purpose. To the best of the authors’ knowledge, no previous studies exist that have attempted to investigate this aspect in the context of pain assessment. We were expecting that this study would produce the same positive feedback as in the usability testing studies mentioned above, and that pain sufferers would similarly embrace the benefits that the 3D pain drawing offers in supporting their everyday reporting of pain.

Accordingly, in order to address the above issues, the main aim of the analysis described in this paper was to identify and report patients’ perceptions of adopting the developed 3D technological solution supporting improved assessment of pain, in the anticipation that technology adoption is best predicted by a patient’s attitudes toward the technology, and perceptions about its usefulness.

## Methods

### Design

**Figure 2:**
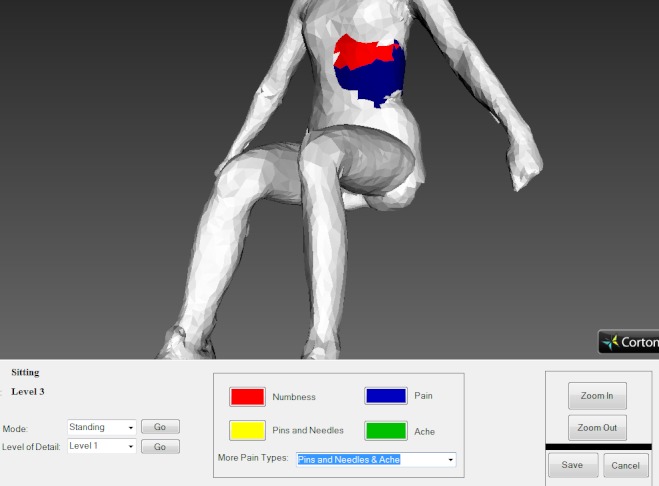
Example screenshot of the 3D pain drawing used.

The above aim was addressed as part of a within-subjects study that we conducted with a convenience sample of 3 different groups of patients [2,27]. Participants from each group of this study were randomly given the 3D pain drawing of Figure 2 and were asked to use it in order to report their pain experience at the moment of use. In order to record their perceptions and understand the patients’ attitudes with respect to the 3D pain drawing being adopted into everyday practice for the assessment of their pain experience, the authors employed a phenomenological approach. Ethical approval was obtained by both the Brunel University Research Ethics Committee and the North London 1 Research Ethics Committee.

### Participants and Recruitment

For the purpose of this analysis, 3 different patient groups were targeted. The first group consisted of spinal cord injury patients (n=15), the second group were patients suffering from rheumatologic pain (n=13), and the third were individuals with some form of back pain (n=32). These participant groups totalled 60 individuals (26 female, 34 male; mean age 47.6 years, SD 12.9), who volunteered to take part in the study.

Recruitment was by convenience sampling—participants were patients in the Royal National Orthopaedic and Northwick Park hospitals, and members of the Hillingdon Independent Wheelchair User Group, all in the greater area of London. In order to approach the participants, we contacted the above organizations’ clinical staff and/or administrators and asked them to recommend a number of people who could potentially satisfy our expected needs, and thus, take part in the study. The criteria for selection was that the participant had a medical condition that involved pain, was 18 years or older, and experienced some pain during the period of the evaluation.

Of the 60 participants who were asked to report their perceptions of using the 3D pain drawing to record their pain experience, the perceptions of only 24 (response rate 43%) were deemed usable and were consequently considered in the subsequent analysis.

### Instrumentation and Data Collection

Prior to collecting any data, informed consent was obtained from all participants. They were all given the 3D pain drawing to evaluate, the results of which are presented in previous work of ours [2,27]. For this study, participants were interviewed one week after using the 3D pain drawing to review their perceptions and attitudes towards adopting the technology to report their pain experience in everyday practice. Participants were not informed about the specific role of the interviewer as being part of the 3D pain drawing’s development team in this study in order to avoid any potential risk of biased results, in the sense that participants could give overly favorable responses.

Semi-structured interviews were chosen for this purpose, as they are considered a valid and consistent method of data collection in phenomenological research. Specifically, participants were offered the opportunity to provide oral comments and/or suggestions about their experiences in assessing their pain using the 3D approach, through the interview questions presented in Textbox 1below.

**Interview questions agenda.**

1. What is your opinion about the pain drawing currently used to report your pain?

1.1. What do you think is the best or worst aspect of this tool, and why?

1.2. What do you think needs most improvement, and why?

2. What is your opinion about the 3D pain drawing you recently used to report your pain?

2.1. What do you think is the best or worst aspect of this tool, and why?

2.2. What do you think needs most improvement, and why?

3. Please state any comments/suggestions that you might have for both tools, if any.

4. What would you want and need from a pain assessment tool?

The above agenda was developed in order to ensure that participants would be able to provide their perceptions through an open-ended discussion that would allow them to focus on better exploring and communicating their thoughts and needs, as well as would help the authors identify issues related to their attitudes and particular needs regarding a 3D pain drawing. The interviews and data collection were conducted by the same person, and lasted approximately 20-30 minutes each, all producing rich information. All responses were recorded using a digital voice recorder, along with notes that would allow for further interpretation of the recorded information.

### Coding and Data Analysis

The responses of each participant were analyzed by employing a qualitative analysis approach, and particularly by combining thematic analysis with the principles of the grounded theory, which is a well-established approach in health care research (eg, [28-31]). Our selection of using the grounded theory lies in its capacity to help researchers formulate hypotheses or theories based on studied phenomena, or to discover participants’ main concerns and how they continually try to resolve it [32]. Along these lines, identifying factors that could support the employment of such 3D technological advancements by patients in the assessment of pain can serve as important findings for further research studies. To discover what these factors are, and considering that there is a lack of prior research in this context, the grounded theory was employed, as it can provide the holistic view necessary to capture patients’ perceptions from which the aforementioned factors could be derived.

Accordingly, thematic analysis and principles of the grounded theory were employed in the following systematic manner. Initially, the recorded interviews were transcribed and coding began by following the process of open-coding, which involves the systematic reading and consideration of every comment produced by each participant, using a line-by-line analysis [33]. The authors then read and reviewed the data, and subsequently developed a coding frame to facilitate the grouping of emerging issues from the data into core themes. This frame suggested the development of a core theme on the basis of grouping codes, which were abstracted from the various participant responses. As such, the data that were formed into core themes were the most relevant to the purpose of this study.

The collected data from each interview were further compared to each other, and to the data produced by the other interviews in order to find any similarities and repetitions, or differences in the emerging issues. Additional grouping codes were added as new issues emerged during the comparison process. Inter-rater reliability scores were not produced since the interviews were not structured [34].

Lastly, all findings were extensively re-reviewed by all authors, in order to validate the data, to gain a high-level understanding of the collected information that would help to identify potential participant attitudes, as well as reflect on any further implications and compare with previous work in the pain literature. All findings were then assessed with a thematic analysis.

## Results

### Overview

The thematic analysis produced 4 main high-level core themes related to the participants’ responses to the interviews, namely: (1) better assessment, (2) perceived clinical utility, (3) intention to use, and (4) perceived ease of use.

The first core theme highlighted the potential that the 3D approach could offer towards the efforts to better facilitate the reporting, and thus, the assessment of the pain experience. A secondary core theme derived further suggested the participants’ perceived clinical utility of the 3D pain drawing by focusing on its capacity to assist them in becoming better stakeholders in the pain management process.

The third and fourth core themes that were identified brought to the fore the participants’ intentions to use the 3D pain drawing in their everyday pain assessment routine, and whether they believed it would be easy for them to systematically interact with, respectively. Table 1 presents a pool of selected participant responses that support these results, which are summarized and elaborated in the discussion that follows.

**Table 1:** Selected participant responses and core themes.

Core theme	Code	Selected responses
**Better assessment**		
	Accuracy	*It (3D approach) allows me to accurately pinpoint any location I choose.* [R1]
	Clarity	*(3D approach) is much better in showing where my pain is...the whole body seems to be closer to reality than the diagram (2D pain drawing) is.* [R2]
	Clarity	*You can actually focus better on that one (3D approach), as the body area is well represented and I can more easily show where my aches are.* [R3]
	Clarity	*The figure (2D pain drawing) was not adequate…I would definitely prefer something better.* [R4]
	Accuracy	*Being able to move it (ie, rotate it) and have a closer peek on my different body parts (ie, zoom in/out) makes me feel that I have a much better control of how to show where my pain is.* [R5]
**Perceived clinical utility**		
	Allows correlation with activities	*It (3D approach) can allow me to better correlate the pain I am experiencing in certain parts of my body with the activities that I had been doing.* [R6]
	Allows correlation with medication	*It (3D approach) made me realize that I was taking my medication at the wrong time of the day.* [R7]
**Intention to use**		
	User enthusiasm	*Amazing! The old one (2D pain drawing) should be retired now.* [R8]
	User preference	*It (3D approach) is more “friendly” to me… I actually prefer this since our body parts are now easier to see and show.* [R9]
	Increases user experience	*The diagram (2D pain drawing) is a bit “cold”, and to be honest, I wouldn’t mind using something better.* [R10]
	User preference	*I prefer something better than that (2D pain drawing) and I think your tool (3D approach) is better to show my pain.* [R11]
	User preference	*Now, I would never go back to the old one (2D pain drawing) again.* [R12]
	User preference	*I prefer your tool instead of the paper (2D drawing) figure.* [R13]
**Perceived ease of use**		
	Ease of use	*Although it might be a bit hard to learn to use, I would like to see this tool (3D approach) again.* [R14]

### Better Assessment

Responses revealed that participants were generally enthusiastic about the level of improvement that the 3D approach offered in assessing their pain experience, which was reported as being significantly better than its predecessor was. Particularly, participants highlighted two important feature advancements of the 3D pain drawing as compared to the 2D one—*accuracy* and *clarity*. The comments that were indicative of this view (Table 1, R1-R5) generally indicated the extent of the 3D approach to cover almost all assessment aspects of the existing conventional 2D pain drawing, while offering an enhanced level of detail. Building on this, participants overly reported that the 3D approach significantly contributed towards the improvement of current pain assessment practices.

### Perceived Clinical Utility

This second core theme revealed that participants were particularly enthusiastic with respect to the ability of the tool to offer a more accurate and structured means of correlating activities and medication intake with pain. It has to be pointed out that the above finding was not a direct result of 3D technology usage, but, was instead identified as an indirect implication of using a 3D tool to report pain.

Specifically, the study revealed that participants were provided the capability to better localize their pain location through 3D technology, whilst concurrently being able to monitor how various activities impacted their pain level at that localized body location. For instance, a participant found that she could manage her pain much better by reducing those activities that led to intense pain at that certain body location (R6).

Moreover, additional evidence came from another participant who claimed that he was also able to better monitor the progression and type of pain, vis-a-vis his prescribed medication/treatment (R7). This observed discrepancy between medication intake and experienced peaks of pain at certain body locations, ultimately resulted in that participant reducing his medication (strong analgesic) by 25%, with no deterioration in the pain levels encountered. Indeed, the reduction of medication intake as a result of self-monitoring of pain was not a singular observation, as this was also reported by 5 other members of our participant group.

### Intention to Use

The evidence produced from participant responses seemed to suggest that a 3D approach to pain assessment was seen as being of important clinical value to the participants. In fact, the majority of them were enthusiastic about the capabilities that the 3D approach provides with respect to better reporting pain to the clinicians involved in the assessment process, and they were generally in line with the response provided by one of the participants (R11) with respect to his preference for the 3D pain drawing to better report his pain experience as opposed to the 2D pain drawing (R9, R11-R13).

### Perceived Ease of Use

The overall responses seemed to indicate that the majority of the participants did not mention or was not concerned with the 3D pain drawing’s potential ease of use. On the contrary, it must be remarked that from the transcribed responses, the general trend was that participants were enthusiastic about the functionality of the 3D pain drawing, with the majority of them expressing their expectations for the 3D drawing to better assist them in more efficiently reporting their pain.

## Discussion

### Principal Findings

As the need for quality health care provision continues to expand, 3D technology can be an efficient and intuitive approach for improving medical practices. The current paper set out to explore a qualitative perspective on the attitudes of 24 patients towards using a 3D pain drawing to report their pain experience, with a focus on understanding their perceptions and needs regarding the enhancement of current pain assessment practices with 3D technological advancements.

The findings from our reported study in the area of pain assessment suggested that participants generally accepted the introduction of a novel 3D approach for the purpose of reporting their pain characteristics as an alternative to the conventional 2D pain drawing, supporting the quantitative findings obtained in previous studies [2,27]. Specifically, the present findings revealed that participants generally appreciated the enhanced ability that the 3D approach offered with regard to reporting their pain. In particular, participants were enthusiastic about the capability it provided to better report their pain characteristics to the involved clinician(s), with results highlighting their preference and satisfaction in using the 3D approach to better describe and evaluate their pain in relation to everyday activities and/or medication received. This has led the authors to hypothesize that its employment in everyday practice could have a medium-term result, with the consequent improvement of the patient-clinician communication channel due to the more comprehensive communications for both parties using the 3D approach to assess pain.

### Comparison with Prior Work

In order to fit our results in the field of current pain research, we will compare our findings with those of previous work in the field. As such, our findings seemed to be consistent with the results of previous studies that looked at the added value of using computerized interfaces for pain assessment among various patients [35-38]. In addition, our study results support past findings regarding the success of 3D technology in the *management* and *treatment* of pain, where 3D technological solutions have been effectively used to treat conditions such as phantom limb pain [39-42], as a distraction technique for burn pain care [43-45], as an effective aid in reducing pain through hypnosis [46-48], and to decrease pain in cancer patients [49-51].

However, our review of the literature has shown that there is a paucity of research investigating the use of 3D technology for the *assessment* of pain, as opposed to past efforts that focused solely on the management and treatment aspects. Therefore, while 3D technology has been extensively used for health data reporting over the past 20 years, we are unaware of any work employing 3D technology for the collection of pain-related data, with the exception of the authors’ previous work.

Consequently, the authors’ view on this study is that 3D technology should not be applied to the above identified problem for the sake of it, but that any new 3D approach should be more intuitive than the existing solutions for pain assessment, and just as usable as the 3D technological tools for pain management and treatment. The hypothesis is that the 3D pain drawing tool presented in this study could offer a significant improvement to the current level of patient pain assessment.

Our study, therefore, provided additional qualitative evidence to further demonstrate the attitudes of patients about the adoption of 3D technology, and supported the above hypothesis by showing the important role that it could also play in *collecting*, *reporting,* and *assessing* their pain experience. The latter has been shown to be highly valued by those with pain-related conditions, as shown by this study’s results.

### Potential Implications

Our findings seem to suggest that employing a 3D approach for pain assessment practices could, in the medium-term, have important implications. Specifically, in line with the authors’ anticipation, our overall findings propose that the successful application of 3D technology in pain assessment could play an important starting role towards improving the provision of quality health care.

In particular, considering that individuals with pain often rely on several health care institutions (eg, hospitals, health, and social care institutes) for assistance, it is anticipated that the 3D pain drawing could be adopted by these institutions for the purpose of assisting the sufferers with their pain reporting by employing a more efficient and accurate pain assessment tool, supporting the use of 3D technology in the overall pain management process.

The capability therefore, offered by the 3D pain drawing is theorized to have significant implications in clinical practice. Even if usability issues are present, as one of the participants suggested (R14), we speculate that most individuals would overlook this, since the convenience factor associated with it would outweigh such considerations (eg, there could be less hospital visits).

The above assumption is also supported by the clinicians involved in the quantitative evaluations of the 3D pain drawings described in past work [27]. According to their comments, the possibility of individuals self-reporting their pain might have very positive implications towards improving the provision of quality care, since individuals could: (1) remotely monitor the progression and type of their pain, vis-a-vis their prescribed medication-a finding also confirmed by the present study’s results, (2) become better stakeholders in managing their pain, and (3) also benefit from a psychological point of view.

From another perspective, it is generally accepted that while the cornerstone to efficient pain management is the successful assessment of the pain experience, this effort often relies on the health care professional’s empathy, interest, and understanding of a patient’s condition at the time of assessment [52]. However, the clinician’s heavy workload or tiredness, for example, could often affect the aforementioned aspects. As such, although the 3D approach does not offer a diagnosis, it could potentially reduce the need for the above constant reliance on the clinician and ease his/her assessment, through its capacity to facilitate better pain self-reporting and remote assessment. This could also potentially ease the congestion and waiting times often experienced in health care settings, and further contribute to reducing hospital visits.

It is therefore speculated that the above could first, empower individuals with pain as a result of receiving improved quality of care, and second, could help moderate the role of health care institutions.

### Limitations and Future Work

Apart from the positive findings, this study has also raised certain limitations and avenues for future work that accordingly need to be acknowledged. First, we acknowledge that our participant sample size was relatively small. Unfortunately, it was rather impractical to recruit a large number of participants for this study, as only individuals with pain could be considered. It would, thus, be beneficial to involve a larger sample, in order to support a more informed study result. Second, it is recognized that the findings of the present study cannot be generalized with respect to every 3D technology used in health care. However, our findings could be used to draw generalized conclusions with regard to the usefulness of 3D models, and further the attitudes of patients towards the adoption of such 3D models in order to record health-related findings. Given the limited existing research efforts in this particular area of health care, our findings may be considerably useful as they could offer significant insight and could be used as an important point of reference for future efforts.

Third, although one of the beneficiaries of this study’s findings is health care providers, the work presented in this study was limited to the perceptions of patients admitted to hospitals. As such, it has to be made clear that since this study was prototypical, it has not yet been tested in the remaining health care settings. In retrospect, an attractive future direction would be the investigation of the 3D approach with patients from the whole range of health care providers. Finally, future research may also pay attention to a wider range of pain-related medical conditions by examining the attitudes of, for instance, patients recovering from surgery or being treated for cancer.

### Conclusions

The findings of this study show that, despite past research efforts, individuals with pain are still not entirely satisfied with the adequacy of current pain assessment practices. As it has been revealed by our findings, this could be due to several factors including the individuals’ limited understanding of their pain and the factors that may or may not affect it, as well as to their inability to accurately describe it to medical professionals using the conventional tools.

Consequently, it has been speculated that the use of 3D technology as an alternative could create the possibility for individuals with pain to become better stakeholders in the management of their pain experience. This in turn could have positive implications towards improving the provision of quality health care, particularly with respect to contributing towards time-effective and improved care for individuals with pain. These findings may be of considerable interest to health care providers, policy makers, researchers, and other parties that might be actively involved in the area of pain research and management.

## Abbreviations

2D: 2-dimensional
3D: 3-dimensional
